# Robotic Evaluation and Repair of Penetrating Injury of the Abdominal Wall

**DOI:** 10.7759/cureus.63685

**Published:** 2024-07-02

**Authors:** Anastasiya Shchatsko, Gabriel Swenson, Andrew Vasyluk

**Affiliations:** 1 Surgery, Central Michigan University College of Medicine, Saginaw, USA; 2 Radiology, Mayo Clinic Scottsdale, Scottsdale, USA; 3 Surgery, Covenant Hospital, Saginaw, USA

**Keywords:** penetrating abdominal injuries, robotic repair, robotic hernia repair, robotic exploration, robotic, robotic abdominal surgery, robotic-assisted exploratory laparoscopy, robotic traumatic hernia repair, robotic surgery for penetrating abdominal injuries, robotic surgery in trauma

## Abstract

This case demonstrated the feasibility of robotic-assisted exploratory laparoscopy in a hemodynamically stable trauma patient and abdominal wall repair with a favorable outcome. The patient presented with a stab wound at the left middle posterior flank. A computer tomography scan of the abdomen and pelvis demonstrated penetrating soft tissue injury to the left lateral abdominal wall with herniation of the omentum. A robotic-assisted laparoscopic approach was implemented to evaluate for visceral injury and to repair the abdominal wall. Diagnostic laparoscopy ruled out visceral and diaphragmatic injuries, and robotic primary tissue repair of the abdominal wall was performed. The patient was discharged home the following day. Laparoscopy for hemodynamically stable trauma patients has shown the benefit of decreased morbidity and decreased hospital stay compared to laparotomy. In turn, the robotic surgical approach has all the benefits of laparoscopy while bringing additional benefits of improved surgical dexterity, visualization, range of motion, and ergonomics.

## Introduction

Penetrating abdominal injuries frequently require exploratory laparotomy: in 71% of gunshot wounds and in a lower number of stab wounds (39%) [1,2]. That is why hemodynamically stable patients with stab wounds, instead of surgical exploration, may require additional imaging studies, local wound exploration, or observation. However, if surgery is indicated, laparoscopy for hemodynamically stable trauma patients has shown the benefit of decreased morbidity and decreased hospital stay compared to laparotomy [3]. That is why the World Society of Emergency Surgery (WSES) consensus suggests a laparoscopy-first approach for stable patients with abdominal trauma [4]. In turn, the robotic surgical approach for hemodynamically stable patients has all the benefits of laparoscopy while bringing additional benefits of improved surgical dexterity with wristed instruments, visualization, range of motion, and ergonomics. This case implemented robotic-assisted exploratory laparoscopy in a hemodynamically stable patient with penetrating trauma with a favorable outcome. The demonstration of this case should increase awareness of the approach and employ its use more frequently. Appropriate use of laparoscopy can spare hemodynamically stable trauma patients from the unneeded morbidity of a laparotomy.

This case report was presented at the podium at the 68th Annual Meeting of the Michigan Chapter of the American College of Surgeons in Traverse City, Michigan, on May 20, 2022.

## Case presentation

A patient in their 20s presented by walking into the emergency room with a single stab wound without active bleeding at the left middle posterior flank that occurred just prior to presentation. A knife of unknown size and type was removed before arrival. The patient was hemodynamically stable with a blood pressure (BP) of 117/72 mmHg, heart rate of 93/min, temperature of 36.7°C, respiratory rate of 20/min, and peripheral oxygen saturation of 99%. The patient’s body mass index was 46, classifying them as class III obesity, posing a challenge to abdominal surgery. The patient had no notable past medical, surgical, family, or psychosocial history. On physical exam, there was localized tenderness around an 8 cm laceration on the left middle posterior flank and no peritoneal signs. In this urgent presentation, there were no financial or access diagnostic challenges.

**Figure 1:**
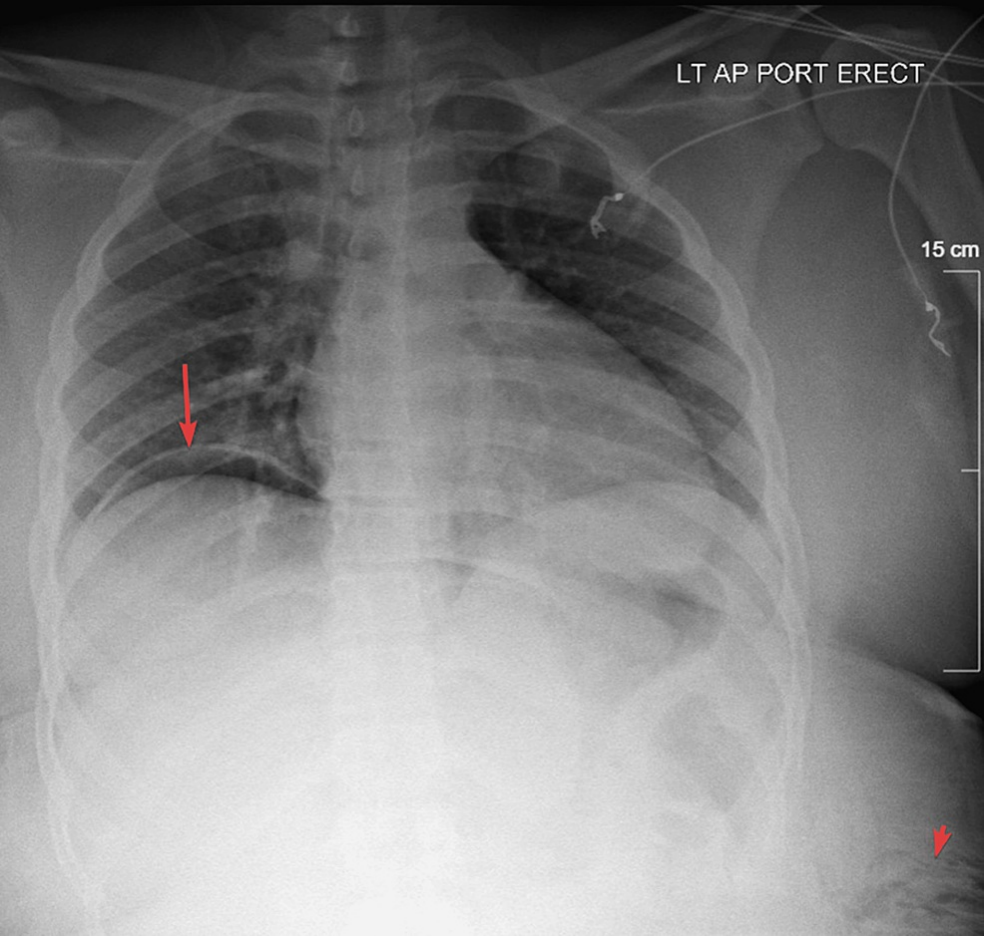
Upright chest X-ray that included the portion of the abdomen. Free intraabdominal air under the right diaphragm is marked with a long arrow, and subcutaneous air in the left middle flank area is marked with a short arrow.

**Figure 2:**
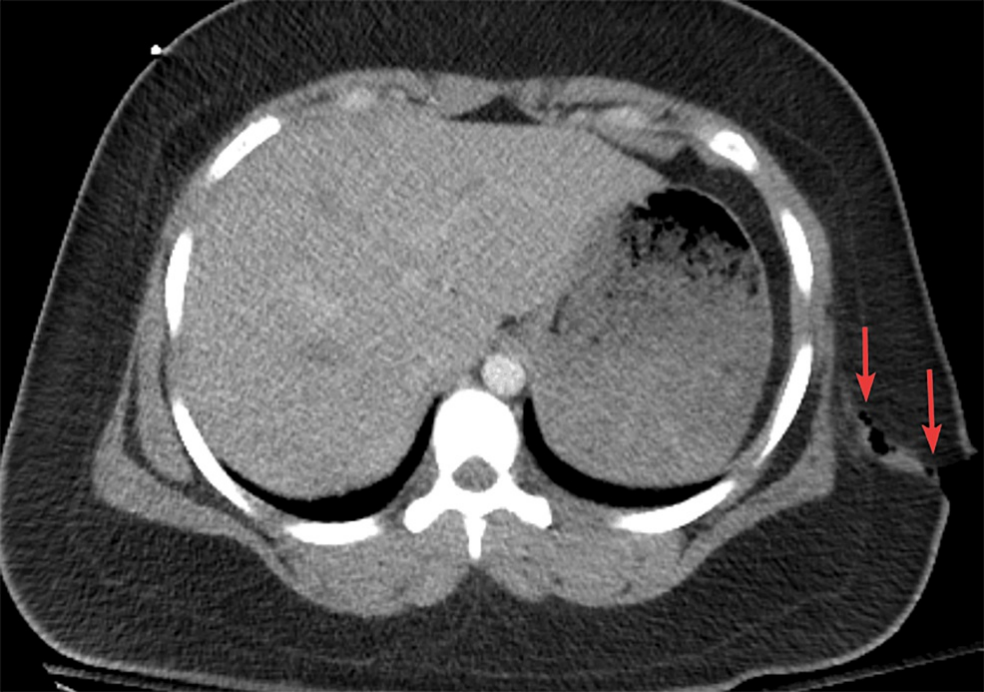
Contrast-enhanced computed tomography, axial view of the penetrating wound through the left flank skin and subcutaneous fat. The wound is marked with an arrow.

**Figure 3:**
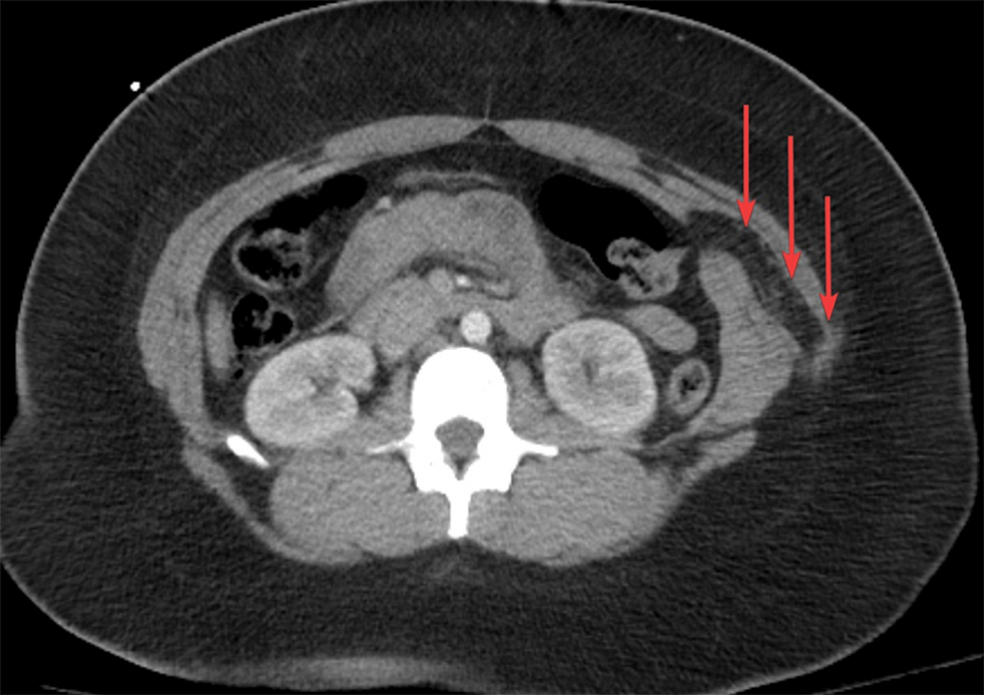
Contrast-enhanced computed tomography, axial view of the penetrating wound through the left-sided abdominal musculature and into the peritoneum with herniation of the omentum. The herniated omentum is marked with arrows.

Upright chest X-ray was negative for pneumo- and hemothorax but revealed subdiaphragmatic free air (Figure 1). The focused assessment with sonography for trauma exam was negative for free intraabdominal fluid. The patient underwent a contrast-enhanced computed tomography scan of the chest, abdomen, and pelvis, which demonstrated no thoracic or visible intraabdominal organ injury; there was a penetrating soft tissue injury to the left flank with oblique trajectory starting from the chest in the posterior axillary line and entering the abdominal cavity with herniation of the omentum (Figures 2-3).

**Figure 4:**
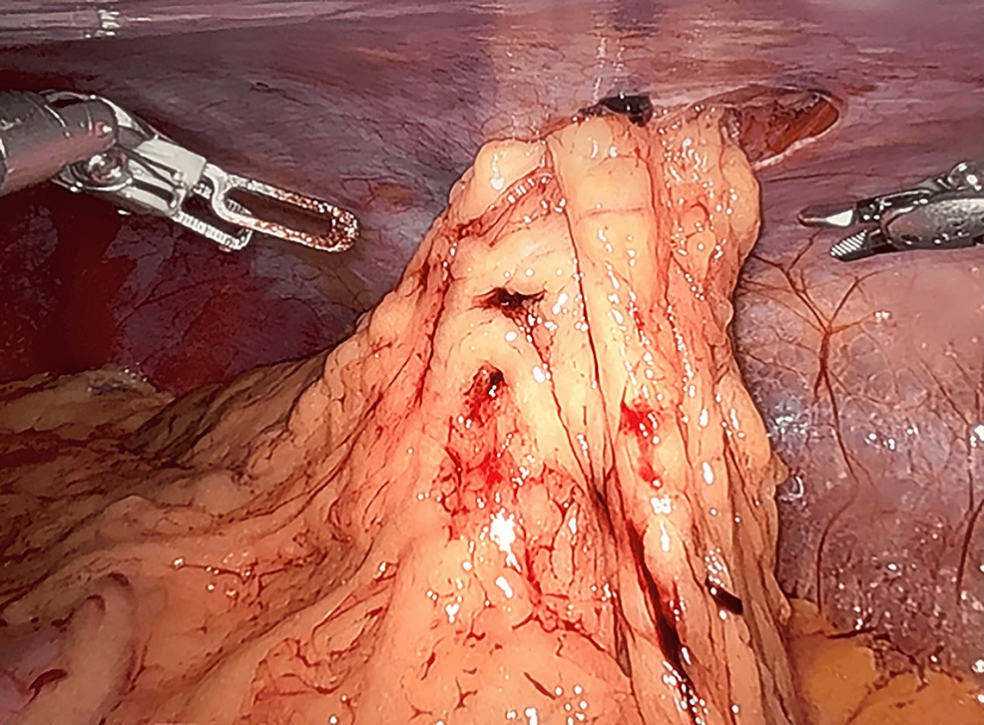
Intraoperative photo of omental fat herniating through the penetrating abdominal wall defect.

**Figure 5:**
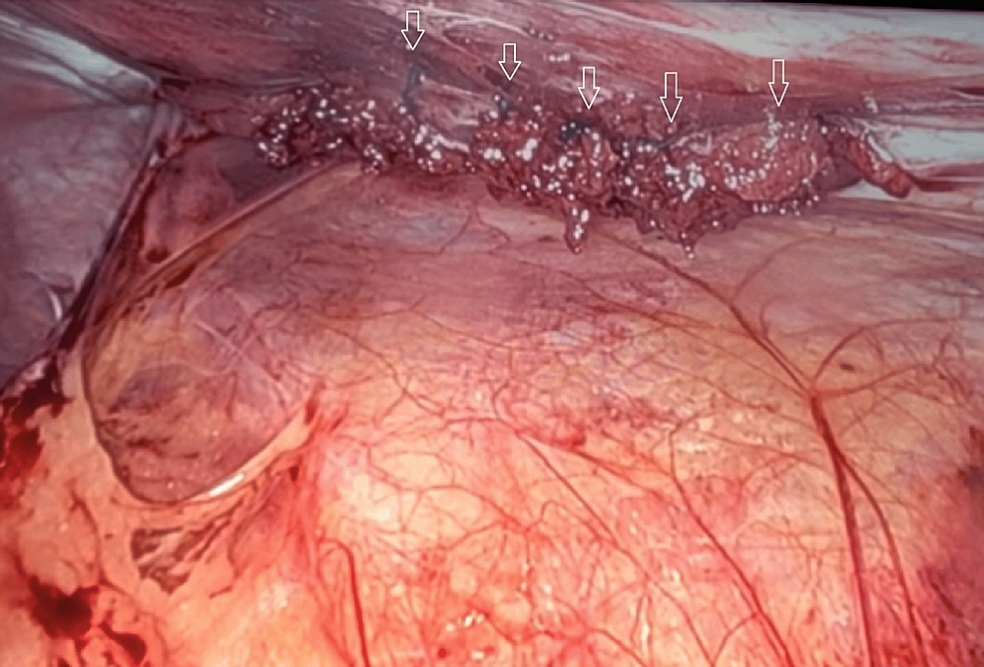
Intraoperative photo after robotic repair of the abdominal wall defect. The suture line of size 0 absorbable barbed self-locking suture (V-Loc™) approximating the flank muscle and fascia is marked with arrows.

To evaluate for intraabdominal injury, as well as repair the abdominal wall, a robotic-assisted laparoscopic approach was chosen (DaVinci - Intuitive Surgical, Sunnyvale, CA). The wound was washed out and closed prior to the insufflation of the abdominal cavity, which allowed for the maintenance of the pneumoperitoneum. Laparoscopy ruled out visceral and diaphragmatic injury and visualized an abdominal wall defect that was 3 x 5 cm and was located just lateral to the semilunar line (Figure 4). Primary tissue repair of the abdominal wall defect was performed with robotic assistance without mesh, given wound contamination. Size 0 absorbable barbed self-locking suture (V-Loc™) was used, taking all the visible layers in the suture line (Figure 5). The case duration was one hour and 23 minutes.

The patient tolerated the procedure well and was discharged the following day in stable condition. The patient was seen one week after the surgery, and they developed a mild post-operative seroma that resolved without intervention. The phone follow-up was done at 12 months post-surgery. Upon questioning, the patient denied experiencing any pain, bulging, or skin changes in the surgical area at the 12-month check.

## Discussion

The patient, presenting with a penetrating abdominal wall injury, was hemodynamically stable. This stability allowed us to exclude potentially life-threatening thoracoabdominal injuries through computed tomography scans of the chest, abdomen, and pelvis. Subsequently, the patient was urgently taken to the operating room for confirmation of no intra-abdominal injury and abdominal wall repair to prevent herniation, obstruction, and strangulation of intraabdominal organs. Given the patient's hemodynamic stability, we anticipated good tolerance of pneumoperitoneum and therefore opted for diagnostic laparoscopy over laparotomy. This approach aimed to avoid the morbidity associated with negative exploratory laparotomy and to reduce the hospital stay. Furthermore, we proceeded with robotic-assisted abdominal wall repair. Given the stab wound's tangential, oblique trajectory in the flank area, coupled with the patient’s morbid obesity and significant subcutaneous fat layer thickness, robotic suturing was favored over open hernia repair due to its lower wound morbidity. An open approach would have necessitated extending the skin incision for improved visualization. Although access to a robotic system may be restricted by institutional availability or the presence of trained staff, and its usage might typically be reserved for elective procedures, we were able to employ the robotic platform for this emergency case. Our institution provides 24/7 access to a robot with trained personnel; additionally, the robot was not in use for other surgeries at that time.

Advantages of diagnostic laparoscopy over laparotomy for hemodynamically stable patients include a reduced risk of acute complications such as deep vein thrombosis, pulmonary embolism, pneumonia, wound infections, wound dehiscence, and long-term complications such as adhesions and ventral hernias [3]. After a laparoscopic approach, the smaller surgical incisions result in less postoperative pain and shorter hospital stays. In contrast, hospital stays following negative laparotomies are longer, with a typical mean length of stay of 4.7 days (median: 5, range: 2-8 days), increasing with complications and associated injuries [5], thereby inflating healthcare costs.

Our case shows that the robotic-assisted laparoscopic approach in a hemodynamically stable patient carries all the benefits of laparoscopy. It additionally offers improved surgical dexterity with wristed instruments, which aids in suturing at difficult angles, provides enhanced three-dimensional visualization, increases the range of motion, and improves ergonomics. The consensus statement from WSES for emergent general robotic surgery recommends strict patient selection, use only in hemodynamically stable patients, and only if expertise is available [4]. It has been demonstrated that robot undocking and conversion to open surgery in cases of life-threatening emergencies can be performed swiftly by adequately trained personnel [6].

We believe that it is feasible to extrapolate data about robotic and laparoscopic approaches from elective hernia repair to traumatic hernia repair. Robotic ventral hernia surgery has been shown to have a safety profile comparable to laparoscopic surgery [7]. Both robotic and laparoscopic approaches are superior to open surgery in terms of pain, morbidity, and length of stay. However, the robotic approach is associated with longer operating times and higher costs. Three randomized controlled trials comparing elective laparoscopic and robotic ventral hernia repairs found no significant difference in outcomes regarding pain, functional status, satisfaction, hospital stays, wound complications, hernia recurrence, and readmission rates [7-9]. Nevertheless, several reports indicate that the robotic approach significantly increases healthcare costs and operative times [7-10]. Conversely, a more recent systematic review and meta-analysis found a shorter length of stay for robotic incisional hernia repair compared to laparoscopic [10]. Additionally, the latest meta-analysis has shown that robotic ventral hernia repairs, compared with laparoscopic, have a lower rate of conversion to open surgery and a lower rate of bowel injuries, while robotic inguinal hernia repair has a lower frequency of recurrences than laparoscopic [11]. Arising evidence from emergency surgery suggests that, for robotic compared to laparoscopic cholecystectomies and appendectomies, the operative times are similar. However, the robotic approach has a lower conversion rate to open surgery and a shorter length of stay [12]. Emergent robotic sigmoidectomy, as opposed to laparoscopic, significantly lowers the conversion rate to open surgery (7.9% vs. 28.7%) and reduces the anastomotic leak rate [13].

Regardless, robotic technology is increasingly adapted for elective ventral hernia repairs, and it is anticipated that, with this increased adoption, costs will eventually decrease. Furthermore, some authors suggest that, if hospitals consider the overall savings from a shorter length of stay and a reduced rate of conversion to open surgery, the cost of robotic surgery could be lower [12].

One of the benefits of robotic suturing over laparoscopic suturing includes a three-dimensional camera with optimal control by the operating surgeon and high robotic camera stability compared to a laparoscopic camera operated by an assistant. This allows for better identification of musculofascial layers, which, in turn, facilitates more precise closure and may potentially decrease pain compared to an en masse closure. Of course, laparoscopic closure could be accomplished with an extracorporeal fascia closure needle, which is a simple and cost-effective tool. However, we believe that it would be technically challenging in our case of a morbidly obese patient with an oblique trajectory of the stab wound in a flank area to identify and close those overlapping fascial layers separately. In addition, an en masse closure would require taking extremely large bites to ensure adequate closure, which may cause significant postoperative pain. In contrast, robotic technology allowed us to better visualize and identify all the layers and to perform a more deliberate small bite closure of overlapping layers with two rows of suture lines. Moreover, with a robotic approach, running the suture is easier than with laparoscopic techniques and allows for even distribution of the tension along the suture line, which might be more durable and could save time. Additionally, robotic suturing does not require a skilled assistant at the bedside since both docking the robot and the suturing itself can be done by a single surgeon.

We believe that the robotic surgery skillset that many surgeons have acquired from elective surgery can be transferred into acute care and trauma surgery. Furthermore, undertaking relatively simple urgent robotic cases is an excellent strategy for surgeons who are beginning to introduce robotics into their practice, as it allows for a safer and smoother transition to more complex emergent cases. After overcoming the learning curve, robotic cases become more time-efficient.

## Conclusions

For hemodynamically stable trauma patients with isolated abdominal wall injuries, robotic abdominal wall repair is a feasible and efficient option. Robotic surgery for stable trauma patients brings all the benefits of laparoscopy, including less pain and a decreased length of stay. This is the first case report in the literature on robotic repair for penetrating abdominal wall injuries, which has the potential to become more widespread.

## References

[1] Sander A, Spence RT, McPherson D, Edu S, Nicol A, Navsaria P (2022). A prospective audit of 805 consecutive patients with penetrating abdominal trauma: evolving beyond injury mechanism dictating management. Ann Surg.

[2] Martin MJ, Brown CV, Shatz DV (2018). Evaluation and management of abdominal stab wounds: a Western Trauma Association critical decisions algorithm. J Trauma Acute Care Surg.

[3] Hajibandeh S, Hajibandeh S, Gumber AO, Wong CS (2016). Laparoscopy versus laparotomy for the management of penetrating abdominal trauma: a systematic review and meta-analysis. Int J Surg.

[4] Sermonesi G, Tian BW, Vallicelli C (2023). Cesena guidelines: WSES consensus statement on laparoscopic-first approach to general surgery emergencies and abdominal trauma. World J Emerg Surg.

[5] Renz BM, Feliciano DV (1996). The length of hospital stay after an unnecessary laparotomy for trauma: a prospective study. J Trauma.

[6] Fabrício Pereira de Almeida T, Esteves Chaves Campos M, Romanelli de Castro P (2022). Training in the protocol for robotic undocking for life emergency support (RULES) improves team communication, coordination and reduces the time required to decouple the robotic system from the patient. Int J Med Robot.

[7] Dhanani NH, Olavarria OA, Holihan JL (2021). Robotic versus laparoscopic ventral hernia repair: one-year results from a prospective, multicenter, blinded randomized controlled trial. Ann Surg.

[8] Olavarria OA, Bernardi K, Shah SK (2020). Robotic versus laparoscopic ventral hernia repair: multicenter, blinded randomized controlled trial. BMJ.

[9] Petro CC, Zolin S, Krpata D, Alkhatib H, Tu C, Rosen MJ, Prabhu AS (2021). Patient-reported outcomes of robotic vs laparoscopic ventral hernia repair with intraperitoneal mesh: the prove-it randomized clinical trial. JAMA Surg.

[10] Peñafiel JA, Valladares G, Cyntia Lima Fonseca Rodrigues A (2024). Robotic-assisted versus laparoscopic incisional hernia repair: a systematic review and meta-analysis. Hernia.

[11] de'Angelis N, Schena CA, Moszkowicz D (2024). Robotic surgery for inguinal and ventral hernia repair: a systematic review and meta-analysis. Surg Endosc.

[12] Rifai AO, Rembetski EM, Stutts LC (2023). Retrospective analysis of operative time and time to discharge for laparoscopic vs robotic approaches to appendectomy and cholecystectomy. J Robot Surg.

[13] Curfman KR, Jones IF, Conner JR, Neighorn CC, Wilson RK, Rashidi L (2023). Robotic colorectal surgery in the emergent diverticulitis setting: is it safe? A review of large national database. Int J Colorectal Dis.

